# Regulated vascular smooth muscle cell death in vascular diseases

**DOI:** 10.1111/cpr.13688

**Published:** 2024-06-14

**Authors:** Zheng Yin, Jishou Zhang, Zican Shen, Juan‐Juan Qin, Jun Wan, Menglong Wang

**Affiliations:** ^1^ Department of Cardiology, Renmin Hospital of Wuhan University, Department of Geriatrics Zhongnan Hospital of Wuhan University, Wuhan University Wuhan China; ^2^ Cardiovascular Research Institute, Wuhan University Wuhan China; ^3^ Hubei Key Laboratory of Cardiology Wuhan China; ^4^ Center for Healthy Aging, Wuhan University School of Nursing Wuhan China

## Abstract

Regulated cell death (RCD) is a complex process that involves several cell types and plays a crucial role in vascular diseases. Vascular smooth muscle cells (VSMCs) are the predominant elements of the medial layer of blood vessels, and their regulated death contributes to the pathogenesis of vascular diseases. The types of regulated VSMC death include apoptosis, necroptosis, pyroptosis, ferroptosis, parthanatos, and autophagy‐dependent cell death (ADCD). In this review, we summarize the current evidence of regulated VSMC death pathways in major vascular diseases, such as atherosclerosis, vascular calcification, aortic aneurysm and dissection, hypertension, pulmonary arterial hypertension, neointimal hyperplasia, and inherited vascular diseases. All forms of RCD constitute a single, coordinated cell death system in which one pathway can compensate for another during disease progression. Pharmacologically targeting RCD pathways has potential for slowing and reversing disease progression, but challenges remain. A better understanding of the role of regulated VSMC death in vascular diseases and the underlying mechanisms may lead to novel pharmacological developments and help clinicians address the residual cardiovascular risk in patients with cardiovascular diseases.

## Graphical abstract

Regulated vascular smooth muscle cell death, including apoptosis, necroptosis, pyroptosis, ferroptosis, parthanatos, and autophagy‐dependent cell death, play a key role in the development of vascular diseases.

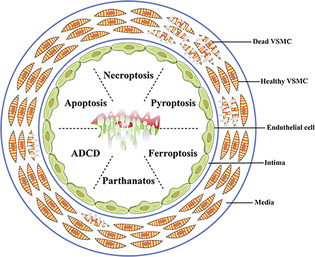

## 1 INTRODUCTION

Cell death has traditionally been divided into two types, regulated cell death (RCD) that requires energy and necrosis that does not. RCD is a regulated process that serves to eliminate unnecessary or potentially dangerous cells in the absence of any external environmental disruption, thus supporting organismal development and homeostasis. However, RCD can also be triggered by intense or prolonged perturbations of the intracellular or extracellular microenvironment, which overwhelm adaptive responses and hinder the restoration of cellular homeostasis. In 1972, Kerr et al. first identified the earliest form of RCD called apoptosis, which is morphologically distinct from necrosis.^1^ Further research has shown that regulated pathways are also involved in a significant portion of necrotic cell death, known as necroptosis.^2^ In addition to apoptosis and necroptosis, several other cell death programs, such as pyroptosis, ferroptosis, parthanatos, autophagy‐dependent cell death (ADCD), entotic cell death, lysosome‐dependent cell death, immunogenic cell death, and NETotic cell death, have been discovered. The mechanisms underlying RCD are diverse and complex across different biological systems. Presently, the dysregulation of RCD is increasingly implicated in vascular diseases.

Vascular smooth muscle cells (VSMCs) constitute the majority of the cellular components in the medial layer of blood vessels and play a crucial role in the performance of the vasculature; they regulate blood vessel tone, blood pressure, and blood flow through contraction.^3^ Unlike skeletal or cardiac myocytes that are terminally differentiated, VSMCs exhibit remarkable plasticity and can undergo reversible phenotypic changes in response to local environmental cues.^4^ In a fully differentiated state, VSMCs exhibit a contractile phenotype, a low proliferation rate, and low synthetic activity, and express specific ion channels and signalling molecules.^5^ However, under pathological conditions, VSMCs transform into a synthetic phenotype characterized by increased proliferation, migration, and production of extracellular matrix (ECM) components.^5^ Together, these findings indicate that VSMCs have significant implications for vascular diseases. However, the regulated forms of VSMC death that occur, evolve and vanish during disease progression remain poorly understood.

**FIGURE 1:**
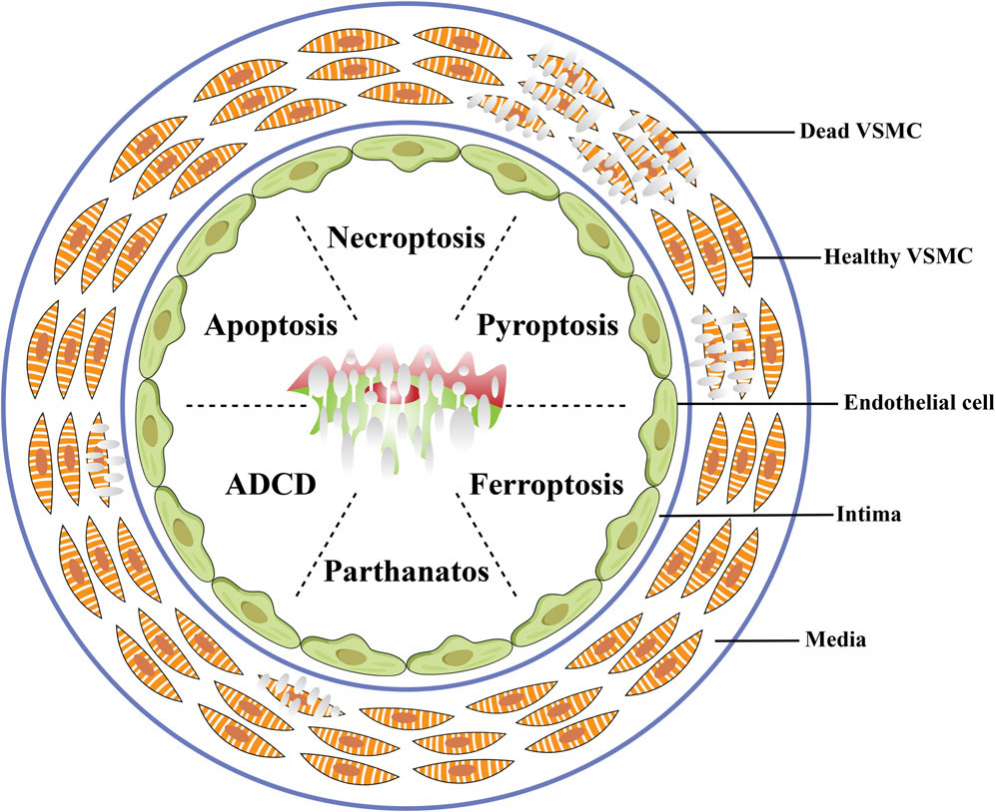
Types of regulated VSMC cell death in vascular diseases. ADCD, autophagy‐dependent cell death.

Herein, we review the current knowledge pertaining to the fundamental mechanisms of regulated VSMC death and the roles of these death programs in major vascular diseases. Emphasis will be placed on apoptosis, necroptosis, pyroptosis, ferroptosis, parthanatos, and ADCD (Figure 1). Entotic cell death, lysosome‐dependent cell death, immunogenic cell death, and NETotic cell death are not discussed in this review. Current evidence does not support the involvement of entotic cell death, lysosome‐dependent cell death, or immunogenic cell death in the death of VSMCs. The effects of NETotic cell death on VSMCs are only indirect, as it primarily affects neutrophils.

## 2 TYPES OF REGULATED VSMC DEATH

### 2.1 Apoptosis

**FIGURE 2:**
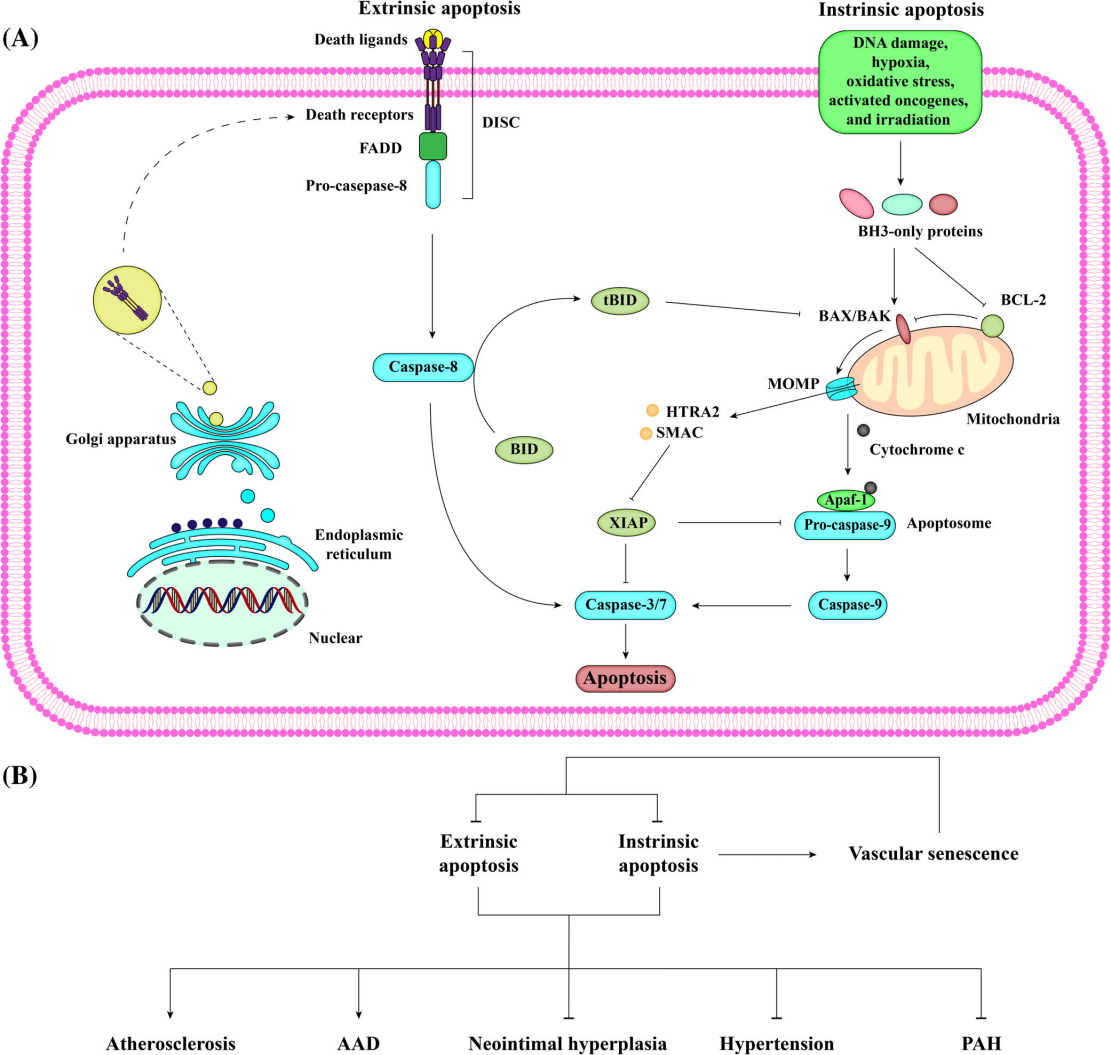
Schematic description of the signalling pathway of apoptosis. (A) The intrinsic pathway is triggered by diverse stimuli, such as DNA damage, hypoxia, oxidative stress, activated oncogenes, and irradiation. These stimuli promote the levels of transcription and post‐translation of BH3‐only proteins. BH3‐only proteins can directly or indirectly antagonize the pro‐survival protein BCL‐2, thereby unleashing BCL‐2‐associated X protein (BAX) and BCL‐2 antagonist/killer 1 (BAK) that mediate mitochondrial outer membrane permeabilization (MOMP). Subsequently, mitochondrial pro‐apoptotic factors, including cytochrome c and SMAC/HTRA2, are released into the cytoplasm. Cytochrome c can bind to apoptotic peptidase activating factor 1 (Apaf‐1), which induces a conformational change in Apaf‐1 that allows it to recruit pro‐caspase‐9, leading to the assembly of a complex termed apoptosome. In this complex, pro‐caspase‐9 is cleaved to form the active caspase‐9 protease, which then activates downstream effector caspase‐3/7. The X‐linked inhibitor of apoptosis (XIAP) inhibits the activation of pro‐caspase‐9 and the function of caspase‐3/7. To allow apoptosis to proceed, SMAC/HTRA2 in the cytosol can bind to XIAP, freeing these caspases. The extrinsic pathway is stimulated by the binding of death ligands to their cognate death receptors (e.g., FasL to Fas). Then, activated death receptors recruit adaptor proteins (e.g., FADD), allowing pro‐caspase 8 recruitment into the death‐inducing signalling complex (DISC). Caspase‐8 proteolytically activates executioner caspases for the downstream of apoptosis. Caspase‐8 also converts the BH3‐only protein BID into its pro‐apoptotic form, tBID, which triggers BAK/BAX‐mediated MOMP and subsequent apoptosis through the intrinsic pathway. Intriguingly, unlike other cell types, VSMCs exhibit resistance to Fas‐induced apoptosis due to the sequestration of the death receptor Fas/TNFR1 in the Golgi apparatus. However, various specific stimuli sensitize VSMCs to Fas‐mediated apoptosis by transporting Fas from the Golgi apparatus to the cell surface. (B) VSMC apoptosis in both intrinsic and extrinsic pathways has been well studied in various vascular diseases, with almost all players identified during apoptosis. VSMC apoptosis promotes atherosclerosis, AAD, and vascular senescence, while preventing intima hyperplasia, hypertension, and PAH. In turn, vascular senescence exhibit resistance to apoptosis.

Apoptosis is the most common type of RCD and is characterized by cell membrane blebbing, cell shrinkage, nuclear fragmentation, chromatin condensation, and chromosomal DNA fragmentation. It can be triggered by two distinct pathways: the intrinsic pathway involving mitochondria, also known as the B cell lymphoma‐2 (BCL‐2)‐regulated pathway, and the extrinsic pathway initiated by death receptors (Figure 2).

Atherosclerosis is a progressive inflammatory disease of large‐ and medium‐sized muscular arteries that typically leads to the formation of plaques and the blockage of blood flow to tissues. The level of apoptosis is initially low in early plaques but increases as lesions develop. Fatty streaks are pre‐atherosclerotic plaques with a cellular‐rich background that is mainly composed of VSMCs. However, these VSMCs in fatty streaks have already exhibit high levels of pro‐apoptotic factors BAX and caspase‐3, which increases the susceptibility of VSMCs to apoptosis.^6^ Fatty streaks can progress to fibroatheromas, where a fibrous cap overlies a lipid‐rich necrotic core. In fibroatheromas, the necrotic core is mainly acellular due to the progressive elimination of VSMCs, while live VSMCs migrate and recover the lipid core to form the fibrous cap. In particular, since VSMCs are the main source of collagen production in the fibrous cap and are responsible for its tensile strength, the loss of VSMCs results in a thin and fragile fibrous cap. Abnormal VSMC loss and collagen deposition induce the rupture or erosion of the fibrous cap, leading to the occurrence of fatal heart attacks, such as myocardial infarction. Moreover, apoptotic VSMCs promote plaque thrombogenicity by exposing phosphatidylserine on the cell surface that can act as a substrate for thrombin, which is detrimental to plaque stability.^7^ Remnants of apoptotic VSMCs in plaques remain as matrix vesicles and can act as nucleating structures for plaque micro‐calcifications, which contributes to plaque development and rupture. In fact, normal arteries have a large capacity to withstand the apoptotic loss of VSMCs.^8^ VSMC apoptosis alone does not induce inflammation, remodelling, thrombosis, calcification, neointima or aneurysm formation in a mouse model of inducible VSMC‐specific apoptosis. In contrast, within established atherosclerotic plaques, acute high‐ or chronic low‐level induction of VSMC apoptosis replicates multiple features of plaque development and putative sequelae.^8^, ^9^ Overall, targeting VSMC apoptosis is a solid strategy for plaque treatment, with early intervention being a better option.

Aortic aneurysm (AA) occurs when the progressive weakening of the aortic wall causes the aorta to enlarge. Aortic dissection (AD) occurs when a tear forms within the aortic wall and causes blood to flow between the laminar layers of the media, thereby separating them and creating a false lumen with a severely weakened outer aortic wall.^10^ Chronic, low‐level VSMC death characterizes AA and dissection (AAD). Severe VSMC apoptosis in AAD tissue is accompanied by an imbalance between BAX and BCL‐2, as well as high levels of p53.^11^, ^12^ VSMC apoptosis is not only a feature of AAD but also a pathological basis for maintaining AAD. The accumulation of apoptotic VSMCs contributes to aortic wall remodelling and early aneurysm development in a murine model of Marfan syndrome, a hereditary connective tissue disorder associated with aneurysm development.^13^ In addition, apoptotic VSMCs release active caspases, including caspase‐2, caspase‐3, and caspase‐7, which act as matrix‐degrading enzymes and lead to elastolysis and ECM remodelling.^14^ In turn, the deterioration of the extracellular environment further triggers extensive apoptosis.^15^ Residential VSMC depletion and coincident ECM degradation make the aortic wall more prone to progressive dilatation and potential rupture. Therefore, targeting VSMC apoptosis represents a significant strategy for the treatment of AAD.

Acute vascular injury occurs in numerous important clinical situations, such as plaque rupture, thrombosis, and therapeutic procedures, such as angioplasty, stenting, or bypass surgery.^10^ Generally, balloon injury of vessels induces two waves of VSMC apoptosis. The first wave is a rapid burst of apoptosis, typically occurring within hours of injury, which results  in a significant reduction in vessel wall cellularity. In a rat carotid artery model, the expression of apoptotic markers increases as early as 30 min after the injury, peaking at 1 h, but becomes undetectable by 4 h.^16^ Intriguingly, VSMC‐cycle activity initiates within 1–2 days after angioplasty, persists for 10 days, and then decreases.^17^ The second wave of apoptosis occurs between 15 and 45 days after endothelial injury and reaches a maximum at 20 days.^18^ During this wave, VSMC accumulation in the neointima reaches a maximal level at 2 weeks after injury, yet excessive cellular proliferation persists for up to 12 weeks.^19^ It is presumed that the rates of neointimal VSMC death and proliferation are in equilibrium after 2 weeks onward, effectively preventing any further increase in lesion size.^20^ It is also thought that apoptotic VSMCs after balloon angioplasty release signals to activate the dedifferentiation of normal VSMCs for neointimal hyperplasia.^21^ This phenotypic change may represent a survival mechanism for VSMCs in response to neighbouring apoptosis, and the inhibition of apoptosis may disrupt this process.^22^

Hypertension is a systemic disease characterized by persistently elevated blood pressure during the systolic and/or diastolic phase in the systemic circulation.^23^ Hamet et al. first proposed that enhanced DNA replication in VSMCs cultured from the aorta of spontaneously hypertensive rats (SHR) occurs in parallel with an increase in apoptosis.^24^ SHR‐derived VSMCs are more prone to excessive proliferation and apoptosis. The imbalance between apoptosis and proliferation may account for abnormalities in VSMC growth in the arterial wall during hypertension.^25^ In hypertensive vessels, increased apoptosis partially counteracts the rapid VSMC growth, suggesting that VSMC apoptosis may contribute to preventing the development and progression of hypertension.^26^, ^27^ During the early treatment, medial VSMC apoptosis in the thoracic aorta of SHR is stimulated by angiotensin‐converting enzyme (ACE) inhibition, AT1 receptor antagonism, or calcium channel blockade, which occurs before the inhibition of DNA replication and is independent of blood pressure reduction.^28^ ACE inhibition restores the susceptibility to apoptosis through the activation of BAX.^29^ Additionally, the pro‐apoptotic and growth‐inhibitory effects of AT1 antagonists require the presence of the AT2 receptor.^30^ The early phase of aortic mass regression in losartan‐treated SHR is characterized by an acute and rapid event, which is synchronized with a transient increase in the BAX to BCL‐2 ratio, caspase‐3 activation, and VSMC apoptosis.^31^ During long‐term antihypertensive therapy, the increase in the apoptotic rate of VSMCs persists through weeks of treatment with ACE inhibition and calcium antagonism.^29^, ^32^ Additionally, another beneficial consequence of apoptosis might be to limit detrimental vasoconstriction.^9^

Pulmonary arterial hypertension (PAH) is a complex and progressive disease characterized by elevated pulmonary arterial pressure and subsequent right ventricular failure.^33^ Pulmonary artery smooth muscle cells (PASMCs) are the main component of pulmonary arteries, and their excessive proliferation and resistance to apoptosis contributes to pulmonary vascular remodelling.^34^ In line with VSMC apoptosis opposing hypertension, enhanced apoptosis in PASMCs could be beneficial for restoring the balance between cell death and proliferation, thereby reversing pulmonary vascular remodelling in severe PAH.^35^ Moreover, the degradation and resorption of excess pathologically deposited ECM, including elastin, synergize with cell removal in this process.^14^

Vascular aging and vascular aging‐related cardiovascular disease manifest as arterial stiffness, degradation, and remodelling, during which VSMCs undergo senescence upon various vascular stimuli such as DNA damage, genomic instability, telomere shortening, and oxidative stress.^36^ During apoptosis, minority MOMP enables the release of mitochondrial DNA into the cytosol, leading to the induction of a chronic senescence‐associated secretory phenotype (SASP).^37^ Therefore, inhibition of sublethal mitochondrial apoptotic stress is a method to prevent SASP. So far, current research has mainly shown that VSMC senescence is accompanied by its apoptosis.^38^, ^39^ Interestingly, senescent cells typically exhibit resistance to apoptosis, driven by upregulation of anti‐apoptotic proteins.^40^ The relationship between VSMC apoptosis and senescence should be further investigated.

### 2.2 Necroptosis

**FIGURE 3:**
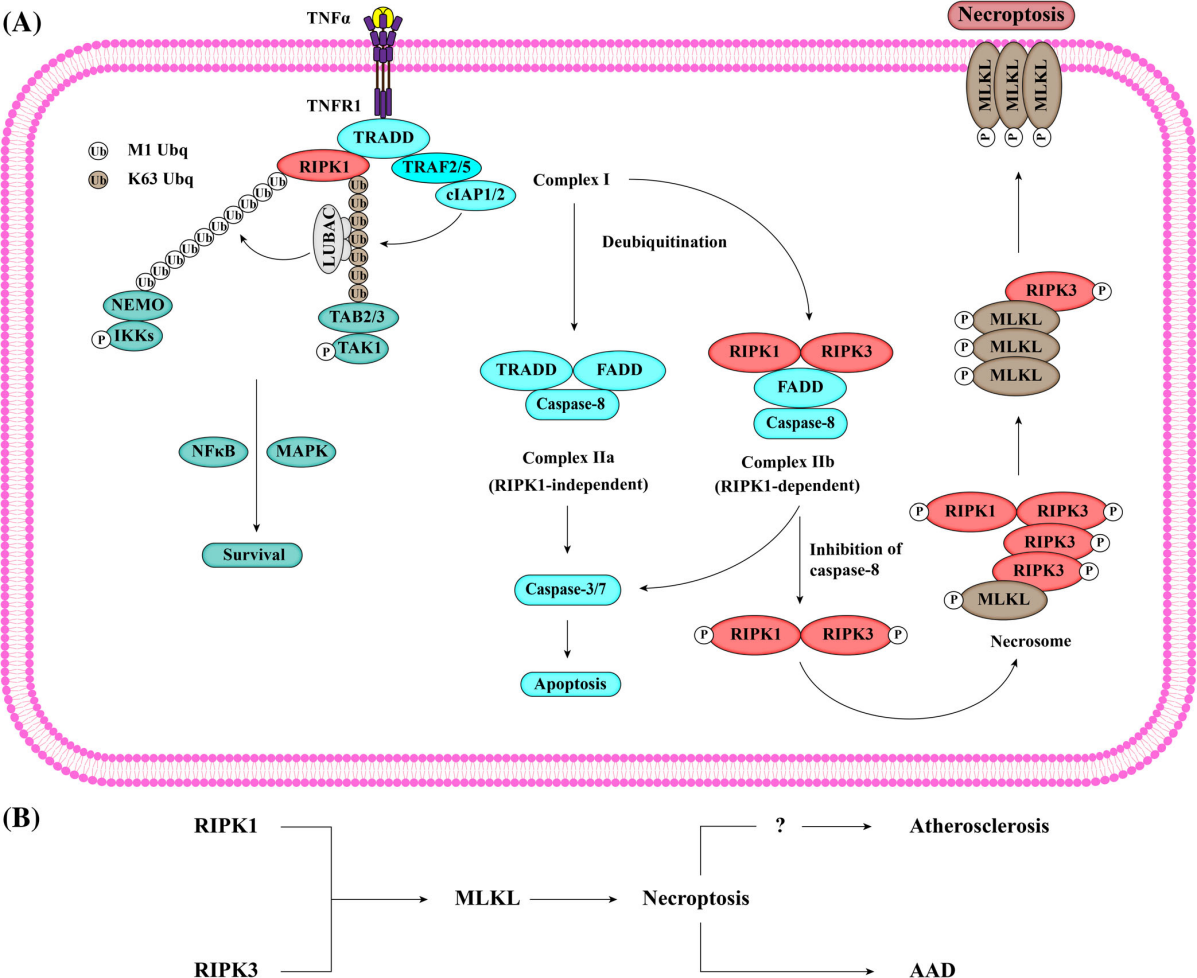
Schematic description of the signalling pathway of necroptosis. (A) Necroptosis is triggered by the binding of death ligands to death receptors (e.g., TNF to TNFR1), allowing the recruitment of TRADD, receptor‐interacting protein kinase 1 (RIPK1), TNFR‐associated factor‐2/5 (TRAF2/5), and cellular inhibitor of apoptosis‐1/2 (cIAP1/2) for complex I formation. Subsequently, cIAP‐1/2 promotes RIPK1 ubiquitination via K63 ubiquitin chains, leading to the recruitment of the linear ubiquitin chain assembly complex (LUBAC). LUBAC generates M1 ubiquitin chains, which are added to RIPK1. Subsequently, M1‐ and K63‐ubiquitin chains serve as scaffolds for the recruitment of the nuclear factor‐κB (NF‐κB) essential modulator (NEMO)‐IκB kinase (IKK) complex and TGF‐β‐activated kinase 1 (TAK1)/TAK1‐binding protein 2/3 (TAB2/3), leading to NF‐κB and mitogen‐activated protein kinase (MAPK) activation. The destabilization of complex I leads to the formation of a second cytosolic complex IIa, which consists of TRADD, FADD and caspase‐8. Under conditions such as TNF stimulation following the loss or inhibition of IAP, a third cytosolic complex IIb, composed of RIPK1, RIPK3, FADD, and caspase‐8, is formed. Activated caspase‐8 within complex II triggers caspase‐3/7 activation, leading to RIPK1‐dependent apoptosis. However, in the absence of caspase‐8 activation, RIPK1 is activated, initiating the necroptosis pathway instead of RIPK1‐dependent apoptosis. This process occurs through autophosphorylation and transphosphorylation of RIPK1 and RIPK3, which recruit mixed lineage kinase domain‐like pseudokinase (MLKL) to induce its phosphorylation and oligomerization. Oligomerized MLKL is then transports to the plasma membrane via the Golgi‐microtubule‐actin machinery, leading to plasma membrane permeabilization, cytokine release, and eventually necroptosis. (B) VSMC necroptosis has been rarely studied in vascular diseases. RIPK1, RIPK3, and MLKL have been identified in VSMC necroptosis during AAD. The relationship between VSMC necroptosis and atherosclerosis is still unclear.

Accumulating evidence has shown that necrosis can be induced in a regular manner in a caspase‐independent fashion, challenging the original perception of necrosis as accidental and unregulated cell death. This type of regulated necrosis is termed “programmed necrosis” or “necroptosis” to distinguish it from necrosis caused by physical or chemical stimuli (Figure 3).

In patients with unstable carotid atherosclerosis, both the mRNA and protein levels of necroptosis mediators RIPK3 and MLKL are elevated.^41^ Phosphorylation of RIPK3 and MLKL, crucial steps in initiating necroptosis, has been detected in advanced plaques.^41^, ^42^ The deletion of RIPK3 or MLKL resulted in a reduction in the size of advanced atherosclerotic plaques in *ApoE* ^−/−^ or *LDLR* ^−/−^ mice but did not affect the early stages of plaque formation.^43^, ^44^ Ox‐LDL triggered the phosphorylation of RIPK3 and MLKL in macrophages, which was dampened by Necrostatin‐1 (Nec‐1), a necroptosis inhibitor. However, necroptosis in VSMCs cannot be induced by ox‐LDL alone or in combination with Z‐VAD‐FMK, a pan‐caspase inhibitor.^41^ This may be because VSMCs uptake modified lipids through different mechanisms than macrophages and may not activate the same signalling pathways in response.^45^ Therefore, further research on VSMC necroptosis in atherosclerosis is needed. Intriguingly, RIPK3 in macrophages and endothelial cells (ECs) protects against atherosclerosis through necroptosis‐independent mechanisms.^46^ The protection mediated by RIPK3 is partially attributed to the inhibition of monocyte chemoattractant protein‐1 in macrophages and E‐selectin in ECs. RIPK1, which lies upstream of the RIPK3‐MLKL necroptotic machinery, acts as a master switch that determines whether a cell undergoes inflammation, apoptosis, or necroptosis in response to extracellular stimuli. Unlike RIPK3 and MLKL, RIPK1 has been identified as a central driver of inflammation in atherosclerosis by promoting the release of inflammatory cytokines through the NF‐κB pathway, which is independent of necroptosis‐associated pathways.^47^

Elevated levels of RIPK3 and RIPK1 in human abdominal AA (AAA), particularly in α‐SMA^+^ VSMCs, indicate the involvement of necroptosis in aneurysm pathogenesis.^48^ RIPK3 signalling was found to contribute to aneurysm pathogenesis in an elastase‐induced AAA model by inducing VSMC necroptosis and stimulating NF‐κB‐mediated vascular inflammation.^48^, ^49^ In a murine CaCl_2_‐driven model of AAA, RIPK3‐mediated VSMC necroptosis involved MLKL and calcium/calmodulin‐dependent protein kinase II (CaMKII), with MLKL phosphorylation occurring upstream of CaMKII.^50^ Pharmacological inhibition of RIPK‐1 with Nec‐1 stabilized pre‐existing aneurysms in an elastase‐induced AAA model by diminishing inflammation and promoting connective tissue repair.^51^ GSK2593074A (GSK'074), a small chemical inhibitor of necroptosis, impeded VSMC death in murine AAA models by targeting both RIPK1 and RIPK3.^52^ In addition, interleukin (IL)‐37 supplementation suppressed RIPK3‐mediated VSMC necroptosis, consequently promoting VSMC dedifferentiation.^53^ Therefore, necroptosis in VSMCs may be a promising target for the treatment of AAA.

### 2.3 Pyroptosis

**FIGURE 4:**
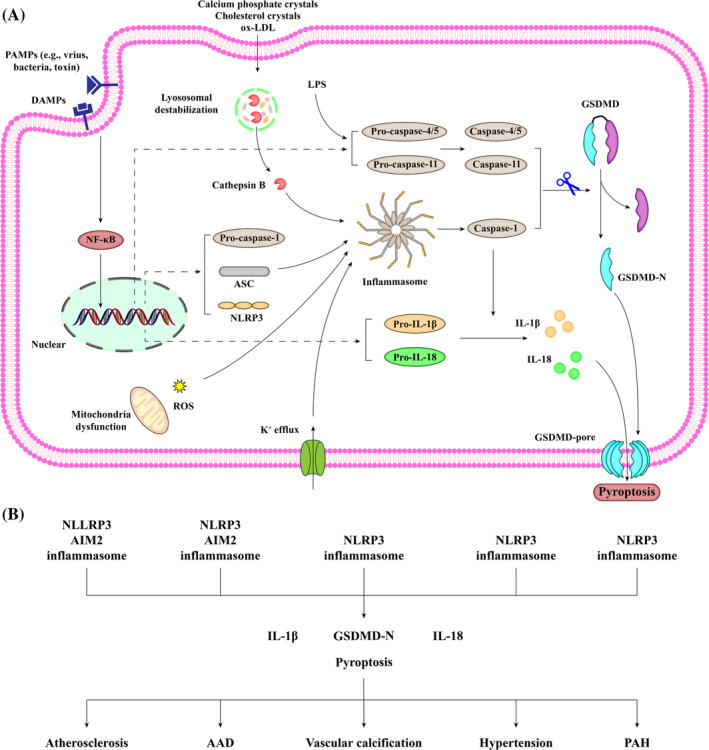
Schematic description of the signalling pathway of pyroptosis. (A) The NOD‐like receptor protein 3 (NLRP3) inflammasome is composed of a sensor NLRP3, an adapter apoptosis‐associated speck‐like protein containing a CARD (ASC), and a effector pro‐caspase‐1. Inflammasome assembly is mediated through the interaction between a pyrin domain (PYD) in ASC and an N‐terminal PYD in NLRP3, as well as the binding of the CARD in ASC to pro‐caspase‐1. This requires two steps: priming and activation. During the priming step, extracellular pathogen‐associated molecular patterns (PAMPs) and damage‐associated molecular patterns (DAMPs) are recognized by PRRs, which upregulates the expression of NF‐κB‐dependent target genes, including NLRP3, ASC, pro‐IL‐1β, pro‐IL‐18, pro‐caspase‐1, and pro‐caspase‐11. The second step, activation, involves various pathways such as potassium efflux, mitochondrial ROS generation, and lysosomal destabilization. After the inflammasome assembly, pro‐caspase‐1 (p45) is activated and hydrolysed into mature cleaved caspase‐1 (p10/p20 tetramer). On the one hand, activated caspase‐1 recognizes and cleaves precursors of IL‐1β and IL‐18 into their mature forms. On the other hand, activated caspase‐1 also cleaves the caustic executor protein GSDMD at the Asp275 site, forming the 22 kDa C‐terminus (GSDMD‐C) and 31 kDa N‐terminus (GSDMD‐N). GSDMD‐N oligomerizes and forms nonselective plasma membrane pores, which promotes the release of inflammatory cytokines, cell swelling, and finally, pyroptosis. In the non‐canonical pyroptosis pathway, the upstream sensory complex caspase‐11 and its human orthologs caspase‐4/5 can directly sense intracellular lipopolysaccharide (LPS) without the need for inflammasomes. Activated caspase‐11 and caspase‐4/5 result in GSDMD cleavage and pyroptosis. (B) VSMC pyroptosis has been identified to promote the development of various vascular diseases, with essential players, such as NLRP3 inflammasome, GSDMD‐N, IL‐1β, and IL‐18. AIM2 inflammation in VSMCs has only been identified during atherosclerosis and AAD.

Pyroptosis is a lytic and inflammatory type of RCD characterized by cell swelling, membrane perforation, and the release of cell contents. The canonical pyroptosis pathway initiates with the assembly of the inflammasome, a large supramolecular complex, resulting in the cleavage of gasdermin D (GSDMD) and the release of IL‐1β and IL‐18 (Figure 4).

The NLRP3 inflammasome is required for atherosclerosis,^54^ and the by‐products of pyroptosis, IL‐1β and IL‐18, contribute to the occurrence and development of plaques.^55^, ^56^ The levels of NLRP3 and caspase‐1 are upregulated in human coronary artery atherosclerosis and are positively associated with disease severity.^57^, ^58^ Plaque VSMCs undergo differentiation into macrophage‐like cells in human atherosclerosis, which is correlated with vascular inflammasome activation.^59^, ^60^ A high percentage of macrophage‐like VSMCs co‐expressing cleaved caspase‐1 and IL‐1β was observed in plaques from symptomatic patients, as opposed to those from asymptomatic patients.^60^ Ox‐LDL can generate an initial signal that is sufficient to activate the NLRP3 inflammasome and induce pyroptosis in VSMCs.^60^, ^61^ Targeting one or more players in the pyroptotic process may be promising for limiting established atheroma and plaque destabilization. MCC950, an NLRP3 inhibitor, or VX‐765, a caspase‐1 inhibitor, significantly blocked IL‐1β processing and VSMC pyroptosis.^58^, ^62^ In addition to NLRP3, another inflammasome sensor, AIM2, is an active participant in atherosclerosis, and its deficiency reduced pyroptosis and plaque formation.^63^ However, contrary to our expectations, the key mediator IL‐1β promoted atheroprotective changes in late‐stage murine atherosclerosis, which was reversed by IL‐1β neutralization or VSMC‐specific loss of *Il1r1*.^64^ Therefore, targeting players in pyroptosis instead of by‐products is a better strategy for inhibiting plaque development.

During the progression of AAD, VSMCs are damaged by mechanical or toxic mechanisms, leading to cell death and subsequent release of necrotic cell debris into the tissue.^65^ In turn, these autologous necrotic cell components are detected by neighbouring VSMCs, triggering an inflammasome response via NF‐κB signalling.^65^ Notably, patients with AAD exhibit significant activation of the NLRP3/caspase‐1 inflammasome cascade, particularly in VSMCs from damaged tissues.^66^ Moreover, this cascade‐mediated pyroptosis directly contributes to the degradation of contractile proteins and biomechanical dysfunction.^66^ Targeting NLRP3 or AIM2 effectively hindered aortic dilatation and prevented the incidence and development of AAD.^67^, ^68^ Disulfiram, a drug used for alcohol addiction treatment, has been identified as an inhibitor of pore formation by GSDMD.^69^ The administration of disulfiram ameliorated pyroptosis in VSMCs and protected against Ang II‐induced AAA in *ApoE* ^−/−^ mice.^70^ However, VSMC‐derived GSDMD exacerbated AAA primarily through non‐pyroptosis‐mediated mechanisms.^71^ One such mechanism involves a reduction in putrescine synthesis due to GSDMD deficiency in VSMCs, resulting in an anti‐inflammatory phenotype and decreased susceptibility to AAA formation in mice.

Vascular calcification is strongly associated with increased morbidity and mortality in patients with atherosclerosis, diabetes, and end‐stage kidney disease. Pyroptosis‐related proteins, such as cleaved caspase‐1, GSDMD‐N, and IL‐1β, are upregulated in calcified VSMCs,^72^ which requires NLRP3 inflammasome activation.^59^ Intracellular ROS activated by high‐calcium/phosphate surrounding VSMCs, is the main contributor to pyroptosis induction.^73^, ^74^ NF‐κB signals, calcium mobilization, and redox imbalance are involved in this process.^75^, ^76^, ^77^ The phosphate burden may activate the Kir channel which sustains the outward current of K^+^ across the VSMC membrane, leading to inflammasome activation and VSMC calcification.^78^ Intriguingly, K^+^ efflux in macrophages that activates NLRP3 inflammasome is mediated by P2X7 receptors.^79^ Evidence indicates that targeting players in pyroptosis,^72^, ^80^, ^81^ blocking NF‐κB signals,^77^ scavenging intracellular ROS,^73^, ^75^ or inhibiting K^+^ efflux,^78^ can prevent the process of pyroptosis in VSMCs, which may provide therapeutic methods for vascular calcification‐related cardiovascular events.

In a 50‐year‐old Finnish cohort, NLRP3 gene polymorphism rs7512998 is associated with systolic and diastolic blood pressure.^82^ Moreover, an increase in the by‐products of pyroptosis, IL‐1β and IL‐18, has been detected in the circulation of hypertensive patients.^83^ The activation of NLRP3 inflammasome was observed in the aorta from SHR and in VSMCs from SHR.^84^ These findings indicate that pyroptosis occurs in VSMCs during hypertension. Interestingly, out of line with apoptosis, pyroptosis predominantly regulates excessive cell growth in hypertension instead of cell death, involving TLR4 and NF‐κB signals in the rapid proliferation of VSMCs in hypertension development.^84^, ^85^ Blocking the upstream of NLRP3 inflammasome activation alleviated blood pressure elevation and hypertensive vascular remodelling in SHR and Ang II‐infused mice.^84^, ^86^, ^87^ Additionally, the knockout of caspase‐1 reversed Ang II‐induced blood pressure elevation and vasodilatory dysfunction.^88^

Inflammasome activation and pyroptosis are correlated with the pathogenesis and progression of PAH. Mice deficient in the inflammasome adaptor proteins NLRP3, ASC, or caspase‐1 demonstrated the attenuation of hypoxia‐induced right ventricular systolic pressure elevating and pulmonary vascular remodelling in models of PAH.^89^, ^90^ Glioma‐associated oncogene family zinc finger 1 (GLI1), a transcriptional activator, promoted pyroptosis in PASMCs through enhancing ASC expression by binding to its promoter, thereby facilitating the development of PAH.^91^ Disulfiram reduced vascular remodelling during PAH by inhibiting GSDMD cleavage and pyroptosis.^92^

### 2.4 Ferroptosis

**FIGURE 5:**
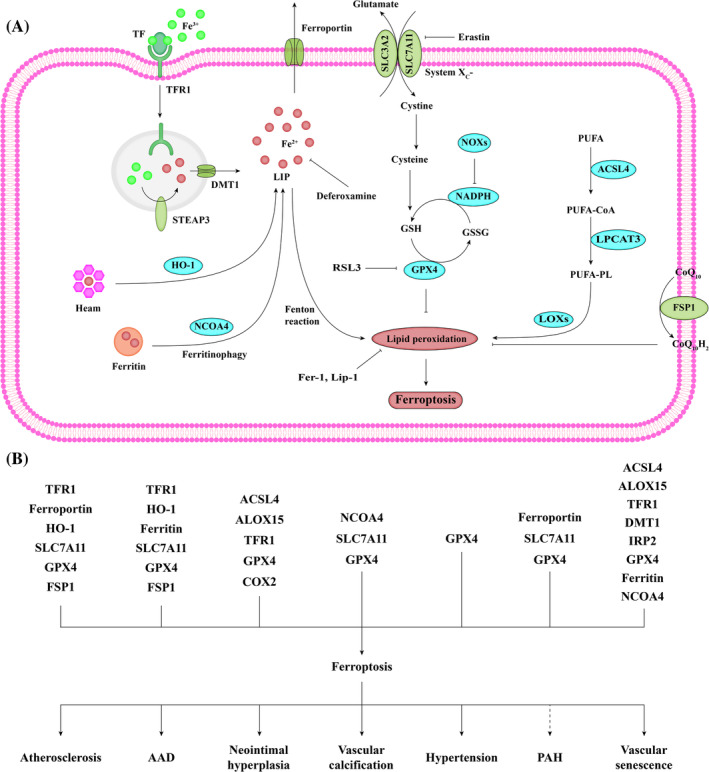
Schematic description of the signalling pathway of ferroptosis. (A) The canonical ferroptosis‐suppressing pathway involves the uptake of cystine via the cystine‐glutamate antiporter (system x_c_‐), which results in glutathione (GSH) biosynthesis. Using GSH as a cofactor, the glutathione peroxidase 4 (GPX4) scavenges the harmful by‐products of iron‐dependent lipid peroxidation, thereby protecting the cell membrane against damage. Ferroptosis is also regulated by the iron metabolism pathway that involves iron uptake by transferrin (TF) and transferrin receptor 1 (TFR1), reduction by the metalloreductase STEAP3, transport by divalent metal transporter 1 (DMT1), storage with ferritin and heme, utilization for Fenton reaction, and export by ferroportin. Ferritin degradation by nuclear receptor coactivator 4 (NCOA4)‐mediated ferritinophagy and heme degradation by heme oxygenase‐1 (HO‐1) increase the labile iron pool (LIP), which sensitizes cells to ferroptosis via Fenton reaction. In addition, fatty‐acid CoA ligase 4 (LACS4), lysophospholipid acyltransferase 3 (LPLAT3), lipoxygenases (LOXs), and NADPH oxidases (NOXs) are involved in the lipid metabolic pathway for lipid peroxidation and ferroptosis. To prevent excessive ferroptosis, phospholipid peroxidation is also inhibited by the ferroptosis suppressor protein 1 (FSP1)‐coenzyme Q10 (CoQ10) system. Exogenously applied ferroptosis inhibitors include radical trapping antioxidants, such as ferrostatin‐1 (Fer‐1) and liproxstatin‐1 (Lip‐1), and iron chelators, such as deferoxamine. Ferroptosis is triggered by the disruption of cystine supply via erastin‐mediated system x_c_‐ inhibition, or the deficiency of GPX4 activity by RAS‐selective lethal 3 (RSL3). GSSG, glutathione disulphide. (B) Players in VSMC ferroptosis have been identified in different vascular diseases. VSMC ferroptosis promotes the development of atherosclerosis, AAD, neointima, vascular calcification, hypertension, and vascular senescence. In PAH, the role of VSMC ferroptosis has been controversial. ALOX15, arachidonate‐15‐lipoxygenase; COX2, cyclooxygenase 2; IRP2, iron regulatory protein 2.

Ferroptosis, a highly iron‐dependent form of cell death, is induced by a variety of mechanisms (Figure 5). The final executor of ferroptosis is excessive lipid peroxidation, which leads to complete cell death.

Atherosclerosis is aggravated by iron overload, resulting in a pro‐oxidant microenvironment, which promotes foam cell development and plaque vulnerability.^93^ Indeed, the restriction of dietary iron intake or iron chelation with deferoxamine significantly reduced experimental plaque formation.^94^, ^95^, ^96^ Iron mainly accumulates in the arterial membrane, allowing plaque VSMCs to acquire a macrophage‐like phenotype. These iron‐overloaded VSMCs are defective, as illustrated by reduced collagen production, increased numbers of foam cells, and elevated lipid levels. Iron accumulation also creates a pro‐oxidative microenvironment in VSMCs, fostering the development of foam cells and promoting plaque rupture. Ferroptosis is triggered by iron deposition and lipid peroxidation in plaque VSMCs, which contributes to plaque destabilization in late‐stage atherosclerosis.^57^ Among the genes implicated in atherosclerosis‐related ferroptosis, HMOX1, encoding HO‐1, has been identified as a critical pro‐ferroptotic gene.^97^ ZnPP, a HO‐1 inhibitor, completely reversed erastin‐induced ferroptosis in VSMCs.^97^ Ox‐LDL is an initial inducer of ferroptosis in atherosclerosis. Iron accumulation and lipid peroxidation have been observed in ox‐LDL‐incubated VSMCs, which is independent of GSH synthesis and metabolism.^98^ However, the SLC7A11/GPX4 axis is associated with ferroptosis in plaque ECs and macrophages.^99^, ^100^ The ferroptosis inhibitor Fer‐1 enhanced endogenous resistance to lipid peroxidation in VSMCs, ECs, and macrophages, thereby reversing plaque progression.

Although iron is an essential element in the maintenance of VSMC function, aortic iron overload leads to tissue damage due to oxidative stress and inflammation in human and murine AAA.^101^, ^102^ Fer‐1 has been identified to alleviate AAA formation.^103^ There is an increase in the ferroptosis‐related molecules TFR, HO‐1, and ferritin, and a decline in the ferroptosis regulatory proteins SLC7A11, GPX4, and FSP1 in the aorta of AD patients, suggesting the involvement of ferroptosis in VSMC dysfunction and AD development.^104^, ^105^ Cigarette smoke extract markedly induced cell death in VSMCs, but not in ECs, which was completely reversed by Fer‐1, Lip‐1 or deferoxamine.^106^ Ferroptosis inhibition has been reported to attenuate β‐aminopropionitrile‐induced aortic dilation and prevent the development and rupture of AD.^104^, ^105^

Enhanced ferroptosis was observed in carotid arteries from mice with wire injury, with high levels of ACSL4, ALOX15, and TFR1, and low levels of GPX4.^107^ Similar changes were observed in VSMCs treated with platelet‐derived growth factor BB (PDGF‐BB). Instead of inducing VSMC death, ferroptotic stress facilitates VSMC dedifferentiation and phenotypic switch during neointimal hyperplasia. The mechanism is related to the disruption of mitochondrial homeostasis. The ferroptosis inducer RSL‐3 directly triggered VSMC dedifferentiation in the absence of PDGF‐BB, which could be reversed by Fer‐1.^107^, ^108^ Therefore, ferroptosis emerges as a novel target for the treatment of neointimal hyperplasia following vascular injury.

Stimulants for in vitro vascular calcification, including palmitic acid (PA), β‐glycerophosphate, and high‐calcium/phosphate, have been reported to induce ferroptosis in VSMCs.^109^, ^110^, ^111^ The repression of the SLC7A11/GSH/GPX4 axis is the main factor for ferroptosis induction. Iron overload also results in calcium deposition in the aorta.^112^ NCOA4‐facilitated ferritinophagy is a key step of PA‐induced ferroptosis in VSMCs, which was reversed by the activation of peroxisome proliferator‐activated receptor alpha.^113^ Targeting ferroptosis with Fer‐1 or deferoxamine attenuated aortic calcification in rats with chronic kidney disease (CKD). Additionally, Metformin alleviated hyperlipidaemia‐associated vascular calcification through its anti‐ferroptotic effects.^109^ Interestingly, both intravenous iron therapy and oral iron therapy have been applied in CKD patients with anaemia.^100^ Therefore, the appropriate dose of iron supplementation for treatment should be confirmed, and additional clinical studies are needed.

A decline in GPX4 in the aortic media was observed in SHR and hypertensive patients.^114^ High hydrostatic pressure is one of biomechanical forces that contribute to the pathogenesis and development of hypertension and its complications. Under high hydrostatic stress, VSMCs exhibit intracellular and mitochondrial ROS overproduction, iron accumulation, and lipid peroxidation, which can be reversed by Fer‐1.^114^ Adequate GSH protects against matrix remodelling and arterial stiffening, whereas its dysregulation disrupts the proper VSMC‐ECM interaction.^115^ Moreover, insufficient GSH synthesis leads to impaired vascular reactivity.^116^ Therefore, enhancing GSH synthesis is a novel strategy for maintaining vascular function and preventing vascular remodelling.

Iron deficiency is involved in almost 40% of PAH patients, and clinical iron supplementation has been reported to attenuate PAH.^117^, ^118^ Evidence reveals that mice with PASMC‐specific ferroportin dysregulation exhibit PAH and right heart failure, which can be reversed by intravenous iron injection.^119^ However, iron chelation can also attenuate pulmonary vascular remodelling in chronic PAH.^120^ In this case, iron deficiency was regarded as a negative feedback mechanism to maintain pulmonary vasoconstrictive response to hypoxia. Therefore, iron supplementation may be a limited approach, which requires further investigation. A recent study showed that ferroptosis could inhibit excessive proliferation in hypoxic PASMCs, which is consistent with the role of apoptosis in PAH. In that study, SLC7A11 overexpression reduced hypoxia‐induced ferroptosis and increased PASMC proliferation, and these effects were reversed by erastin.^121^ Ferroptosis activation seems to be an option for vascular remodelling; however, its negative effects need to be re‐evaluated, as the by‐products of ferroptosis, such as malondialdehyde and 4‐hydroxynonenal, have been found to contribute to chronic hypoxia‐mediated PAH.^122^

Evidence displays a positive relationship between pro‐ferroptosis signalling and vascular aging/stiffness in human.^123^ Ferroptosis signalling molecules, including ACSL4, ALOX15, TFR1, DMT1, and IRP2, and the anti‐ferroptosis molecule GPX4 were downregulated in replicative senescent VSMCs. Similar changes were observed in aortas of aged mice. Additionally, pro‐ferroptosis signalling triggers senescence via secretome‐dependent and independent manners in VSMCs. Conversely, senescent VSMCs are unable to induce ferroptosis.^123^ Pharmacological or genetic inhibition of pro‐ferroptosis signalling is beneficial for delaying vascular senescence, aging, and stiffness, which is mediated through the regulation of NCOA4‐ferritin ferritinophagy.

### 2.5 Parthanatos

**FIGURE 6:**
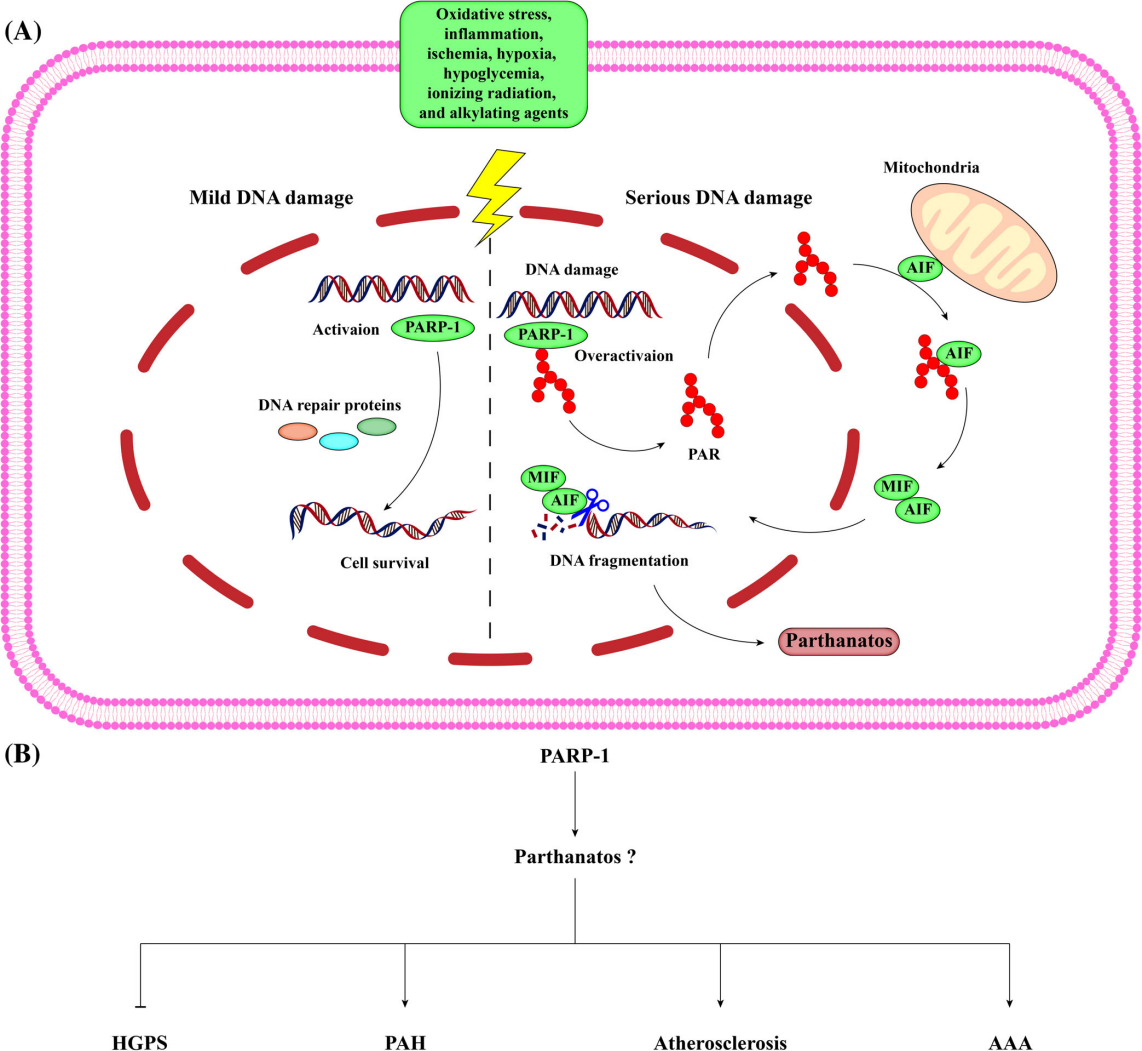
Schematic description of the signalling pathway of parthanatos. (A) Various factors such as oxidative stress, inflammation, ischemia, hypoxia, hypoglycaemia, ionizing radiation, and alkylating agents can cause DNA fragmentation, which in turn activates PARP‐1. When DNA damage is mild, PARP‐1 is activated and recruits DNA damage repair proteins to facilitate the repair process. However, in cases of severe DNA damage, PARP‐1 becomes overactivated, leading to the synthesis of long‐chained and branched poly (ADP‐ribose) (PAR) polymers. These PAR polymers accumulate and are then released from the nucleus into the mitochondria, where they bind to apoptosis‐inducing factor (AIF). Subsequently, AIF is released from the mitochondria and translocated to the cytoplasm. In the cytoplasm, AIF forms a complex with macrophage migration inhibitory factor (MIF), a cytokine with nuclease activity. Subsequently, this complex translocates to the nucleus, triggering large‐scale DNA fragmentation, chromatin condensation, and cell death. (B) VSMC parthanatos has been rarely studied in vascular diseases. The best studied player, PARP‐1, has been identified to plays a role in different vascular diseases, which can provide clues for vascular parthanatos.

Parthanatos is a type of RCD initiated by poly (ADP‐ribosome) polymerase 1 (PARP‐1) (Figure 6). PARP1 is an enzyme that maintains genomic stability by facilitating DNA repair mechanisms, thus promoting cell survival. Meanwhile, it also plays a role in parthanatos in response to extensive DNA damage.

Hutchinson‐Gilford progeria syndrome (HGPS) is a severe human premature aging disorder caused by a lamin A mutant named progerin and is characterized by VSMC loss in large vessels.^124^ Progerin accumulation significantly suppresses PARP‐1, resulting in prolonged mitosis of VSMCs and subsequent death due to mitotic catastrophe.^125^ These findings indicate that PARP‐1 functions as a central regulator of caspase‐independent VSMC death in HGPS.

Increased sustained DNA damage accompanied by PARP‐1 activation has been observed in the distal pulmonary artery from patients with PAH.^126^ Another clinical study showed that the levels of key factors involved in parthanatos, such as PARP‐1, PAR, AIF, and MIF, were significantly increased in PAH patients, particularly in non‐survivors,^127^ which underscores the connection between vascular parthanatos and PAH pathogenesis. The downregulation of miR‐223 triggered PARP‐1 overexpression, resulting in an imbalance between proliferation and apoptosis in PASMCs.^128^, ^129^ The inhibition of PARP‐1 abolishes DNA repair, leading to decreased proliferation and increased apoptosis in PASMCs, thereby reversing Sugen‐ or monocrotaline‐induced PAH.^126^ Therefore, targeting PARP‐1 in the parthanatos pathway may be a novel strategy to prevent rapid VSMC growth and restore the function of pulmonary arteries.

In human atherosclerotic lesions, PARP‐1 is localized exclusively in the nuclei of VSMCs, and its expression is positively correlated with alterations in mitochondrial morphology,^130^ which suggests that the pro‐oxidant environment of the plaque may damage mitochondria through a PARP‐1‐mediated mechanism.^131^ The inhibition of PARP‐1 interferes with plaque development and favours atherosclerotic plaque stability.^132^, ^133^ Within an in vitro system that mimics plaque dynamics, PARP‐1 loss has been shown to improve VSMC viability and protect against cell death induced by H_2_O_2_, TNF‐α, or 7‐KC.^134^ However, the established features of parthanatos in these studies were not rigorously examined, as most work seemly hypothesized that parthanatos preceded cell death.^135^ Therefore, whether parthanatos occurs within VSMCs and kills them in the injured artery remains unclear.

PARP‐1 inhibition not only affects VSMC survival but also changes VSMC physiologic function. The phenotype switching of VSMCs was downregulated by PARP‐1 deficiency via signal transducer and activator of the transcription 1 and runt‐related transcription factor 2, which attenuated atherosclerotic calcification and plaque burden in diabetes.^136^ Moreover, PARP‐1 deletion significantly suppressed VSMC migration, proliferation, and apoptosis induced by Ang II, thereby alleviating the formation and development of Ang II‐induced AAA.^137^ These results may be attributed to the decreased level of parthanatos in VSMCs.

Given the dual role of PARP‐1 in vascular diseases and the limited investigation of parthanatos specifically dependent on PARP‐1 overactivation in VSMCs, it is crucial to conduct further research on vascular parthanatos.

### 2.6 Autophagy‐dependent cell death

**FIGURE 7:**
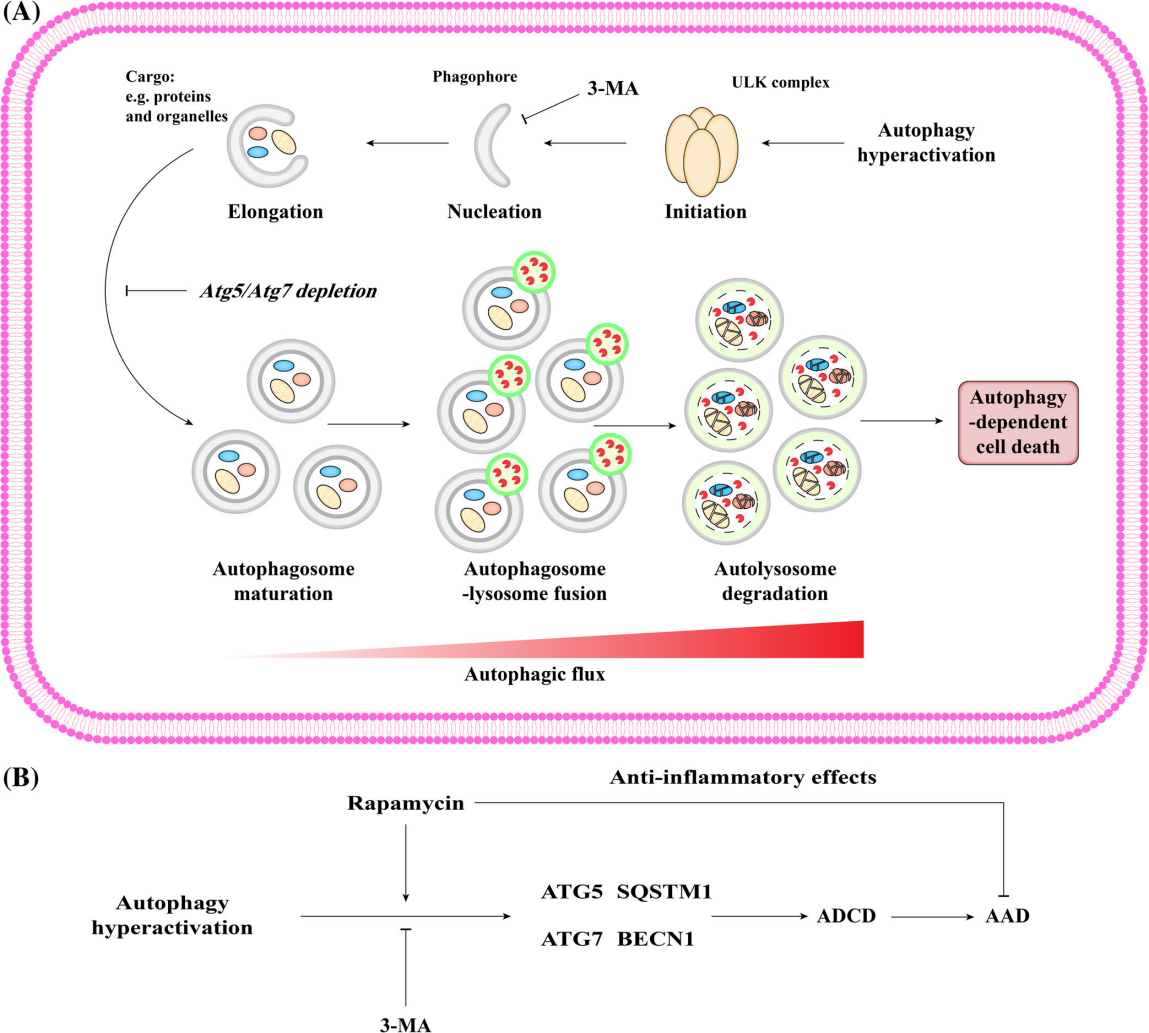
Schematic description of the signalling pathway of autophagy‐dependent cell death (ADCD). (A) The autophagy pathway includes initiation, nucleation, elongation, autophagosome maturation, autophagosome‐lysosome fusion, and autolysosome degradation. ADCD is dependent on autophagy machinery. ADCD is believed to be triggered by autophagy hyperactivation, for example, in response to treatment with natural compounds and specific cancer drugs. An increase in the number of autophagosomes and autolysosomes promotes autophagic flux, which leads to the excessive degradation of unselective cargo and cell death. Since ADCD is dependent on the autophagy machinery, genetically or chemically targeting essential autophagy genes and proteins inhibits the induction of ADCD. (B) Autophagy‐dependent VSMC death has been only identified in AAD, during which players ATG5, ATG7, SQSTM1, and BECN1 are required. 3‐Methyladenine (3‐MA) inhibits ADCD. Rapamycin promotes ADCD, while exert its anti‐inflammatory effects on AAD.

Autophagy is the process by which cytoplasmic material of endogenous or exogenous origin is delivered to the lysosome for degradation for cell survival. However, in specific contexts, the molecular machinery for autophagy etiologically leads to cellular demise. ADCD is a distinct form of cell death that occurs independently of other RCD modalities, such as ferroptosis, necroptosis, and Fas‐driven extrinsic apoptosis.^138^ ADCD generally occurs under pathological conditions due to a failure to preserve cellular homeostasis, which has been poorly understood in VMSCs during vascular diseases (Figure 7).

Osteopontin, one of the most highly expressed genes in AAA, significantly increases the expression of autophagy‐related genes and the formation of autophagosomes, resulting in ADCD rather than cell survival.^139^ The pharmacological stimulation of autophagy by rapamycin exacerbated OPN‐induced aberrant VSMC death, whereas the inhibition of autophagy by 3‐MA produced the opposite results. However, recent evidence indicates that rapamycin can limit the growth of established AAA and prevent the formation of AAD in mice.^140^, ^141^ Most likely, rapamycin may limit AAA through anti‐inflammatory effects rather than through autophagy‐mediated mechanisms. The epigenetic regulation of autophagy essential proteins has been identified to trigger autophagy‐independent VSMC death. Low levels of H3K27me upregulated the expression of ATG5 and ATG7, ultimately leading to ADCD of VSMCs.^142^ Moreover, low levels of H3K9me also facilitated autophagosome formation in VSMCs via directly upregulating SQSTM1 and BECN1 expression, which accelerated autophagic VSMC death.^143^ It is likely that ADCD‐induced VSMC depletion contributes to a reduction in cellularity and the repair dysfunction of ECM. However, evidence showed that VSMC‐specific *Atg5* deficiency increased the incidence and severity of AAA in Ang II‐infused mice,^144^ whereas VSMC‐specific *Atg7* deficiency improved adverse aortic remodelling but not AAA development.^145^ Therefore, targeting autophagy has dual effects on the treatment of AAD.

The degree of autophagy is thought to be the main factor determining whether autophagy is protective or detrimental. However, the appropriate extent of autophagy activity and the threshold for overactivated autophagy to trigger cell death have yet to be determined. Thus, further investigation is needed on the role of ADCD in vascular diseases, which could aid in improving the therapeutic regimes.

## 3 SYSTEM OF RCD

RCD occurs through a variety of subroutines that cause cells to be dismantled in different ways, hence producing distinct morphological changes and immunological consequences. While much of the research thus far has focused on the molecular mechanisms underpinning individual types of cell death, it is now evident that the different RCD pathways do not operate in isolation. In fact, concerning the varying possibilities of triggering and rewiring RCD signalling cascades, all kinds of RCD modalities constitute a single, coordinated cell death system in which one pathway can flexibly compensate for another.^146^

**FIGURE 8:**
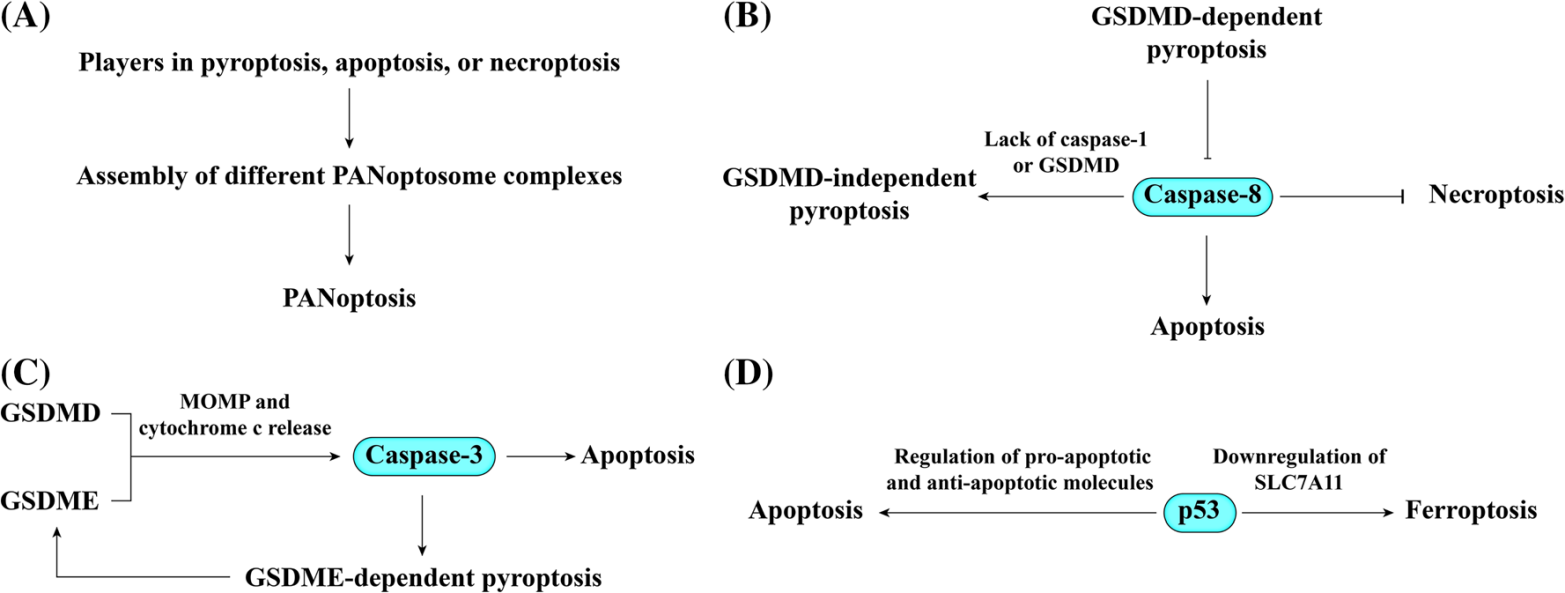
Interaction of RCD modalities. (A) Players in pyroptosis, apoptosis or necroptosis can be integrated to assemble different PANoptosome complexes, which leads to PANoptosis. (B) Caspase‐8 serves a pivotal role by promoting apoptosis and inhibiting necroptosis. Moreover, in the absence of caspase‐1 or GSDMD, caspase‐8 can trigger GSDMD‐independent pyroptosis, but its activity can be counteracted by GSDMD‐dependent pyroptosis. (C) Caspase‐3 induces apoptosis and GSDME‐dependent pyroptosis, with its activation mediated by gasdermin‐induced MOMP and subsequent cytochrome c release. (D) p53 functions as a crucial regulator in promoting apoptosis through modulation of pro‐apoptotic and anti‐apoptotic molecules, while also playing a role in promoting ferroptosis via the downregulation of SLC7A11.

In general, different RCD modalities can share the same stimulus and interact with each other. Some stimuli can simultaneously trigger pyroptosis, apoptosis, and necroptosis in a process, which has been recently termed PANoptosis. PANoptosis functions through the assembly of different PANoptosome complexes that integrate components of the individual cell death pathways in a multiprotein complex that includes caspases, RIPK1, RIPK3, and ZBP1^147^ (Figure 8A). PANoptosis features were observed in the aorta form patients with AAA, mice with AAA, and Ang II‐treated VSMCs.^148^ Moreover, there are several molecular switches between RCD modalities. For example, active caspase‐8 promotes apoptosis, and simultaneously cleaves RIPK1 and RIPK3, thereby preventing MLKL activation and necroptosis induction^149^ (Figure 8B). Moreover, caspase‐8 can also be activated at the inflammasome and subsequently drive GSDMD‐independent secondary pyroptosis in the absence of caspase‐1 protease activity.^150^ However, GSDMD‐dependent pyroptosis prevents caspase‐8 activation at the inflammasome.^150^ Caspase‐3 is another molecular switch between apoptosis and pyroptosis. Activate caspase‐3 is the executioner caspase of apoptosis, and can also cleave gasdermin E (GSDME) to liberate the GSDME‐N domain that targets the plasma membrane to induce pyroptosis^151^, ^152^ (Figure 8C). Gasdermin pores in turn permeabilize mitochondria to activate caspase‐3 during apoptosis and inflammasome activation.^153^ Moreover, the tumour suppressor p53 promotes apoptosis by upregulating mediators of apoptosis, such as BAX,^154^ and downregulating anti‐apoptotic molecules, such as BCL‐2,^155^ and also sensitizes cells to ferroptosis by downregulating SLC7A11,^156^ a subunit of the system x_c_‐, and consequently decreasing cystine uptake (Figure 8D). These observations underline the fine interplay between different RCD pathways.

In this system, a specific RCD modality can induce other RCD subroutines. For example, plasma membrane pores can be formed to induce potassium efflux during apoptosis or necroptosis, which is a known activator of NLRP3 inflammasome.^157^ A different form of interaction occurs when a specific RCD modality triggers RCD in other cells or cell types. For example, dying VSMCs might recruit innate immune cells, such as macrophages, which in turn release cytokines that induce other modalities of RCD and amplify vascular injury.^158^ The regulated death of innate immune cells, such as pyroptosis of macrophages, might result in the release of inflammatory mediators and the activation of immune response, which leads to another type of regulated VSMC death. Additionally, neutrophil‐associated NETotic cell death has been shown to induce extracellular histone H4‐mediated membrane VSMCs.^159^ Although physiological communication between ECs and VSMCs is crucial in vascular diseases, there has been no direct relationship between regulated death of these two cell types so far.^160^

## 4 TREATMENT CHALLENGES

Normal vessels have a strong capacity to withstand relative hypocellularity due to VSMC loss. One the one hand, viable VSMCs are mobilized for self‐growth, migration, and modulate phenotypic transition, which helps to restore the environment where VSMCs are lacking. However, the adjustable capacity of normal VSMCs is limited. Intrinsic compensatory mechanisms do not lead to entire recovery even if RCD is stopped. On the other hand, vascular stem/progenitor cells can also be mobilized in response VSMC loss. Both circulating and resident vascular stem/progenitor cells are capable of differentiating into VSMCs.^161^ An alternative potential cell source for vascular self‐renewal is adipose‐derived mesenchymal stem cells.^162^ However, whether and how regulated VSMC death triggers the proliferation and differentiation of stem/progenitor cells has been not clarified. The debris produced by dead VSMCs can be cleared by neighbouring cells. Phagocytes clear apoptotic cells by binding to phosphatidylserine on the membrane of apoptotic VSMCs. This process, termed efferocytosis, leads to the production of pro‐resolving mediators such as IL‐10, TGF‐β, and specialized pro‐resolving lipid mediators.^163^ However, inefficient removal of VSMC‐derived debris in turn results in secondary necrosis, inflammatory cytokine production, and increased vascular injury.

Given the limitations of intrinsic self‐adjustment, therapeutic approaches for targeting RCD are needed. Multiple small molecule inhibitors targeting RCD have been proven to be effective in the treatment of vascular diseases (Table 1). During the progression of the disease, distinct lethal subroutines act either in a parallel or hierarchized fashion.^164^ Additionally, it is difficult to estimate the true contribution percentage of VSMCs undergoing different RCD types. Therefore, pan‐cell death inhibitors that act on two or more pathways appear to increase the likelihood of success. However, this intervention not only modulates the process in the intended VSMC targets but also modulates cell death in unintended targets, such as inflammatory cells, fibroblasts, and ECs, which is a safety hazard. Instead of therapies targeting all RCD modalities, it is more logical to focus on the predominant mode of regulated VSMC death. Unfortunately, the suppression of apoptosis by caspase‐8 inhibition activates necroptotic pathways, and similar backup systems might function when other RCD modalities are inhibited. Therefore, targeting the correct pathway rather than the predominant form of cell death or main cellular target may be preferred.

**TABLE 1:** Existing small molecule inhibitors targeting RCD.

Small molecule inhibitor	Target	RCD type
Z‐VAD‐FMK	Pan‐caspase	Apoptosis
Necrostatin‐1	RIPK‐1	Necroptosis
GSK2593074A (GSK'074)	RIPK1 and RIPK3	Necroptosis
MCC950	NLRP3	Pyroptosis
VX‐765	Caspase‐1	Pyroptosis
Disulfiram	GSDMD	Pyroptosis
Ferrostatin‐1	Lipid peroxidation	Ferroptosis
Liproxstatin‐1	Lipid peroxidation	Ferroptosis
Deferoxamine	Labile iron pool	Ferroptosis
3‐Methyladenine	Nucleation	ADCD

Strategies for targeting RCD may vary depending on the type and stage of the disease.^146^ Acute vascular damage, such as balloon injury, drives severe VSMC loss mainly through regulated death programs. In fact, most vascular diseases are chronic rather than acute. During the progression of chronic disease, the type, level, and activity of RCD may evolve over time. Early and advanced plaques, for example, exhibit different modalities and levels of RCD. The long‐term inhibition of apoptosis might not be an attractive approach to fight chronic diseases such as atherosclerosis given the likelihood of promoting other more harmful cell death routes.^165^ Therefore, the timing of intervention is particularly relevant for prevention or treatment. Concerning various types of cells undergoing RCD in vascular disease, cell‐specific drug delivery is a promising method for achieving precise treatment. Clinical medication needs further investigation.

There are still many unanswered questions about targeting RCD. How can we prevent the initial wave of cell death and its subsequent consequences? Does preventive intervention still work after RCD has occurred? Should the interference strategy evolve as the disease progresses? What is the predominant RCD target in advanced stages of the disease? How do interventions accurately target the intended VSMCs rather than other unintended cells? Further research is necessary to determine the optimal timing and approach for targeting RCD in human vascular diseases.

## 5 CONCLUSIONS

In this review, we discussed the roles and fundamental mechanisms of regulated VSMC death in major vascular diseases. Various forms of RCD, including apoptosis, necroptosis, pyroptosis, ferroptosis, parthanatos, and ADCD, contribute to the dismantlement of cells in different ways, leading to distinct morphological changes and pathological consequences. These RCD mechanisms collectively form an RCD system in which all kinds of RCD modalities interact with each other. However, several mysteries remain to be resolved regarding the regulated death of VSMCs, such as underlying biology, causes and consequences, and therapeutic manipulations of cell death pathways. Further research is needed to unravel this mystery and develop effective treatment options for vascular diseases.

## AUTHOR CONTRIBUTIONS

Zheng Yin, Jishou Zhang, and Zican Shen contributed to the design and conception of the review article. Juan‐Juan Qin, Menglong Wang, and Jun Wan were responsible for the financial support, and all authors approved the final version.

## CONFLICT OF INTEREST STATEMENT

The authors declare no competing interests.
